# Gut Microbial Stability is Associated with Greater Endurance Performance in Athletes Undertaking Dietary Periodization

**DOI:** 10.1128/msystems.00129-22

**Published:** 2022-05-17

**Authors:** Matthew J. W. Furber, Gregory R. Young, Giles S. Holt, Simone Pyle, Glen Davison, Michael G. Roberts, Justin D. Roberts, Glyn Howatson, Darren L. Smith

**Affiliations:** a School of Life and Medical Sciences, Department of Psychology and Sport Sciences, University of Hertfordshire, Hertfordshire, United Kingdom; b Faculty of Health & Life Sciences, Northumbria University, Newcastle upon Tyne, United Kingdom; c Hub for Biotechnology in the Built Environment, Northumbria University, Newcastle upon Tyne, United Kingdom; d Biomedicine West Wing, Newcastle University, Newcastle upon Tyne, United Kingdom; e Water Research Group, School of Environmental Sciences and Development, Northwest University, Potchefstroom, South Africa; f University of Kent, Canterbury, United Kingdom; g University of Reading Whiteknights, Reading, United Kingdom; h Cambridge Centre for Sport and Exercise Sciences, School of Psychology and Sport Science, Anglia Ruskin University, Cambridge, United Kingdom; Vanderbilt University

**Keywords:** human microbiome, recovery, endurance running, high-protein diet, carbohydrate loading diet, randomized controlled trial, bacteria, bacteriophages, gut microbiome, endurance, physical

## Abstract

Dietary manipulation with high-protein or high-carbohydrate content are frequently employed during elite athletic training, aiming to enhance athletic performance. Such interventions are likely to impact upon gut microbial content. This study explored the impact of acute high-protein or high-carbohydrate diets on measured endurance performance and associated gut microbial community changes. In a cohort of well-matched, highly trained endurance runners, we measured performance outcomes, as well as gut bacterial, viral (FVP), and bacteriophage (IV) communities in a double-blind, repeated-measures design randomized control trial (RCT) to explore the impact of dietary intervention with either high-protein or high-carbohydrate content. High-dietary carbohydrate improved time-trial performance by +6.5% (*P* < 0.03) and was associated with expansion of *Ruminococcus* and *Collinsella* bacterial spp. Conversely, high dietary protein led to a reduction in performance by −23.3% (*P* = 0.001). This impact was accompanied by significantly reduced diversity (IV: *P* = 0.04) and altered composition (IV and FVP: *P* = 0.02) of the gut phageome as well as enrichment of both free and inducible *Sk1virus* and *Leuconostoc* bacterial populations. Greatest performance during dietary modification was observed in participants with less substantial shifts in community composition. Gut microbial stability during acute dietary periodization was associated with greater athletic performance in this highly trained, well-matched cohort. Athletes, and those supporting them, should be mindful of the potential consequences of dietary manipulation on gut flora and implications for performance, and periodize appropriately.

**IMPORTANCE** Dietary periodization is employed to improve endurance exercise performance but may impact on gut microbial communities. Bacteriophage are implicated in bacterial cell homeostasis and have been identified as biomarkers of disequilibrium in the gut ecosystem possibly brought about through dietary periodization. We find high-carbohydrate and high-protein diets to have opposing impacts on endurance performance in highly trained athlete populations. Reduced performance is linked with disturbance of microbial stasis in the gut. We demonstrate bacteriophage communities are the most sensitive component of the gut microbiota to increased gut stress following dietary manipulation. Athletes undertaking dietary periodization should be aware of potential negative impacts of drastic changes to dietary composition on gut microbial stasis and, in turn, endurance performance.

## INTRODUCTION

Elite athletes often follow strict training and diet regimes to maximize performance. Despite decades of research, the ongoing debate around optimal nutrition persists. It is well established that carbohydrate utilization and exercise performance are intrinsically linked (1, 2). For example, carbohydrate is the preferred substrate during high intensity endurance exercise and athletes who consume a high carbohydrate diet (HCD) can maximize endurance performance (2, 3). However, in some circumstances reducing carbohydrate intake during periods of low intensity endurance training can enhance performance via metabolic and cellular adaptations associated with oxidative phosphorylation (4, 5). Dietary periodization is a concept that uses different regimes to optimize the balance between training adaptation and performance. Athletes employ low carbohydrate diets during physical training with the aim of enhancing adaptations (6) before switching to HCD prior to competition to maximize energy stores, thereby improving potential for athletic performance (7).

The implementation of high-protein diets (HPD) during training have become increasingly popular regimes among athletes (8, 9), in part to offset carbohydrate-depleted states. Body mass and body composition are additional factors to consider in endurance performance and HPDs are used during training to help manage these (10, 11) and stimulate muscle growth (12, 13). Despite the potential advantages of HPD, the impact on gut homeostasis is an area of contention (14). In particular, the effects of HPDs on metabolism and gene expression of both gut microbes and host epithelial cells raise questions about longer-term health.

HPDs, especially those rich in red meat, contain large amounts of sulfated amino acids such as cysteine and methionine (15, 16). Proteolytic fermentation of these amino acids in the distal colon is associated with microbial metabolites such as ammonia, phenols, and hydrogen sulfide (17) which can cause damage to the gut microstructure (18) or mucosa (19). Some phenolic compound products of protein metabolism have also been linked with increased gut permeability (20–22). Over prolonged periods, these physiological changes may lead to negative health outcomes. The gut microbiota plays an important role in colonic protein fermentation (23), and high dietary protein consumption has been associated with enrichment of anaerobic bacteria with proteolytic capabilities (24).

Importantly for well-trained athletes, following strict diets and rigorous training regimes, the gut microbiota is implicated in host physiological functions. Endurance exercise is linked to an increased bacterial diversity in the gut due to elevated stress levels (25). Similarly, there are differences in gut bacterial communities in physically active compared to sedentary humans (26, 27). However, these studies do not account for different diets between the populations. This issue has been previously highlighted (26) given that athletes consumed more total energy, fat, carbohydrate, and protein in comparison to less active controls. To date, there are no studies that have explored the microbial communities within the gut across microbial kingdoms in relation to dietary regimens in well-trained athletes. This is of importance given previous work has illustrated the influence of bacteriophage (the viruses of bacteria) as biomarkers of disequilibrium in gut communities (28). Phages are intrinsically linked to the metabolism, stress, and activity of active bacterial communities in the gut. Furthermore, Cronin et al. (32) identified the most descriptive part of the gut microbiota composition of sedentary participants taking on the “couch to 5 km” running challenge was the inducible viral fraction. Despite the potential influence of the viral component, this remains an understudied facet of gut microbial communities, particularly in relation to diet manipulation in highly trained athletes and the influence on human performance.

Previous work has numerous confounding factors of age, lifestyle, diet, physical activity, and other baseline characteristics. This study focuses on a homogenous group of well-trained runners who share baseline dietary and physical activity habits. The aim of this study was to assess the impact of a high-protein versus high-carbohydrate diet on performance in highly trained endurance runners while measuring associated gut bacterial, viral, and fungal communities. Recruiting such a well-controlled cohort enables us to highlight changes to exercise capacity and associated microbial features associated specifically with dietary interventions. Critically to the aim of this study, diet was rigorously controlled which enabled data to be attributable to the manipulation of HCD and HPD.

## RESULTS

### High carbohydrate and high-protein diets have opposing effects on time trial to exhaustion performance.

**FIG 1:**
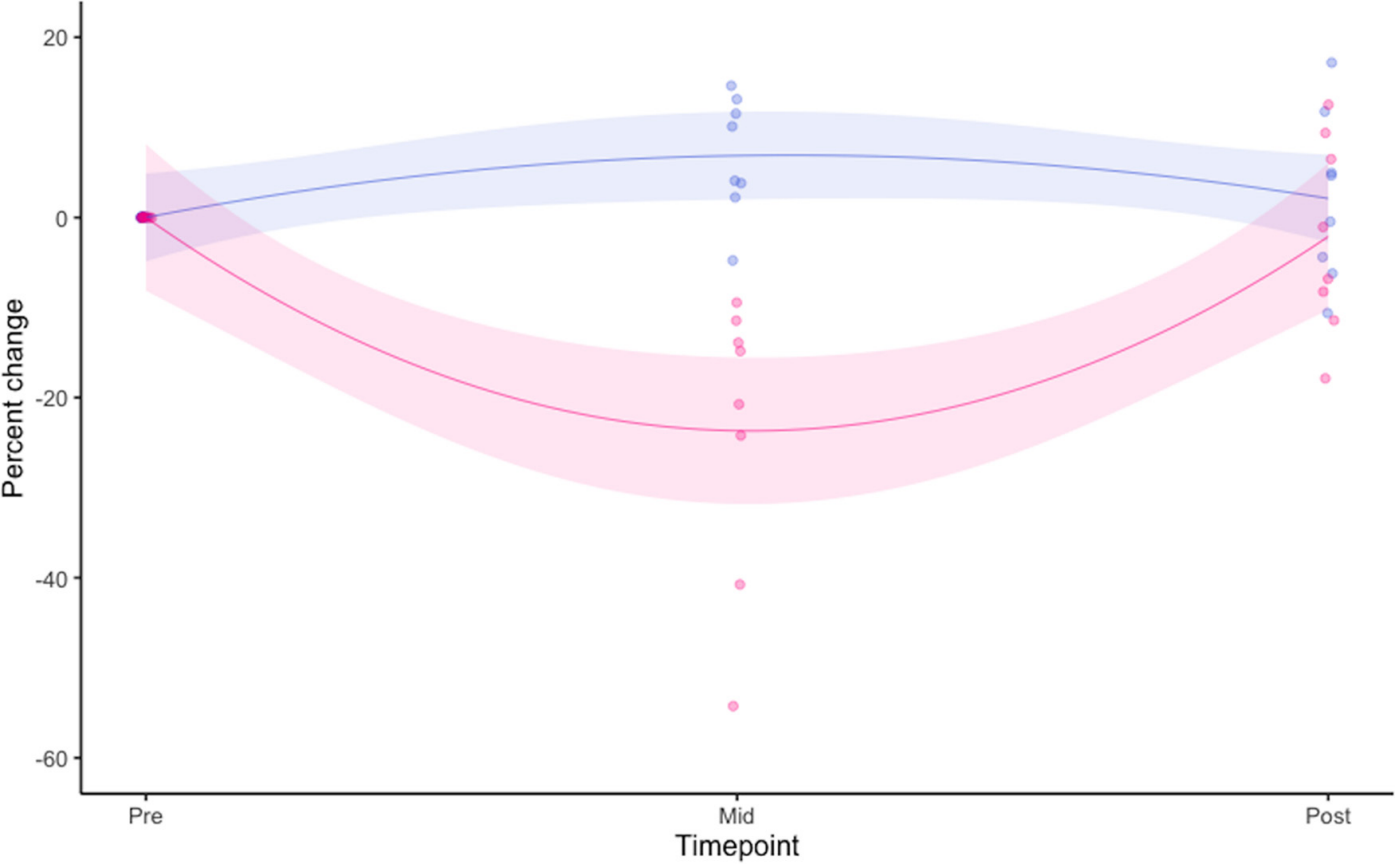
Illustrates longitudinal subject performance during the Max SE trial. Each point represents an individual subject through preintervention (pre), midintervention (mid), and postintervention (post), sampling time points, colored by intervention group (HCD = blue; HPD = pink). The line represents best fit of the linear regression model based on all subjects at that time point. The shaded area describes the 95% confidence intervals of the linear model.

The results of the 95% MaxSE trial are shown in Fig. 1, expressed as relative change (%) from preintervention baseline. Further detail on the impact of the dietary intervention on performance has been previously reported (29). Following HPD, time trial to exhaustion (TTE) was reduced within group by −23.3% midintervention, with all participants exhibiting decreased ability to perform (*P < *0.001). Upon subsequent return to habitual dietary intake, TTE for HPD postintervention time points was comparable with preintervention performance (pre:128.3 ± 29.3 s, mid: 98.4 ± 31.8 s, post: 125.3 ± 32.39 s). In contrast, a 6.5% improvement in TTE was observed midintervention in the HCD group (*P = *0.05) with seven of the eight participants improving relative to preintervention performance. TTE performance for HCD also returned to preintervention levels by trial closure (pre: 182.2 ± 44.4 s, mid: 194 ± 45.5 s, post: 184 ± 38.9 s).

### Poor performance in HPD is associated with changes to microbiota observable in stool.

A total of 48 stool samples from 16 participants (eight HPD, eight HCD intervention), were sequenced to investigate the bacterial, viral, and fungal microbiota. Each participant provided three samples capturing pre-, mid-, and postintervention gut microbiota (see supplementary materials for details of sequencing parameters).

Information on sample sizes and comparisons of samples and controls is available in the supplementary materials (Fig. S1). Significant dissimilarity was observed between controls and samples (*ANOSIM*: R^2^ = 0.99; *P* = 0.001; Fig. S1).

10.1128/msystems.00129-22.2 FIG S1 Library sizes of bacterial (a), FVP (b), IV (c), and fungal (d) samples. Each point represents an individual sample. Library size is depicted on the y axis. Samples are arranged in increasing size order along the x axis. Due to the vast majority of fungal (d) samples containing <100 targeted sequence reads per sample this microbial kingdom was not included in further analysis. FVP (b) and IV (c) samples were included despite mean sample sizes of 234.5 ± 31.2 and 186.6 ± 35.4, respectively, because they were derived from metagenomic datasets. This contrasts with bacterial and fungal read counts which were derived via targeted sequencing of single genes (16S rRNA & ITS regions). Stacked bar chart representing individual sample compositions (e) (sequencing negative = NUNEG; sequencing positive = NUPOS). Composition of individual bacterial samples was significantly different to that of controls when communities were compared by ANOSIM (R^2^ = 0.99; *P* = 0.001). Blocks are colored by bacterial Phylum. The sequencing negative control yielded only 1,911 sequencing reads compared to 29,914 in the next lowest library size. Due to significant dissimilarity between controls and samples, all bacterial OTUs were retained for downstream analysis. Sequencing control samples were removed from the dataset. Download FIG S1, PDF file, 0.1 MB. Copyright © 2022 Furber et al. 2022 Furber et al. https://creativecommons.org/licenses/by/4.0/ This content is distributed under the terms of the Creative Commons Attribution 4.0 International license.

Before comparing impact of dietary interventions on microbial communities we sought to define the baseline of bacterial diversity within the participants enrolled in this study. We compared beta-diversity between participant stool samples in this cohort to age-matched males enrolled in the human microbiome project (30). Significantly lower inter-individual variability was observed between participants enrolled within this study than those enrolled in the HMP cohort (Adonis PERMANOVA *P* = 0.001, Fig. S2). These data suggest that the highly trained athletes enrolled here harbor significantly distinct gastrointestinal bacterial communities to that of a typical western population.

10.1128/msystems.00129-22.3 FIG S2 Beta-diversity was significantly lower between participants of this study and those enrolled in the Human Microbiome Project when assessed by Adonis PERMANOVA (*P* = 0.001). Results illustrate that the baseline characteristics of participants enrolled in this study are more similar than those of participants in the HMP. HMP sequencing data was obtained using the “HMP16SData” package on R, installed using BiocManager. To ensure comparability to the methods employed this study the total HMP dataset was subsampled to include only stool samples collected from male participants on their primary visit and sequenced targeting the V4 region of the 16S rRNA gene. Finally, all OTUs contributing < 0.5% total abundance were removed before calculation of per-sample Bray-Curtis dissimilarity and comparison of community compositions. Download FIG S2, PDF file, 0.04 MB. Copyright © 2022 Furber et al. 2022 Furber et al. https://creativecommons.org/licenses/by/4.0/ This content is distributed under the terms of the Creative Commons Attribution 4.0 International license.

**FIG 2:**
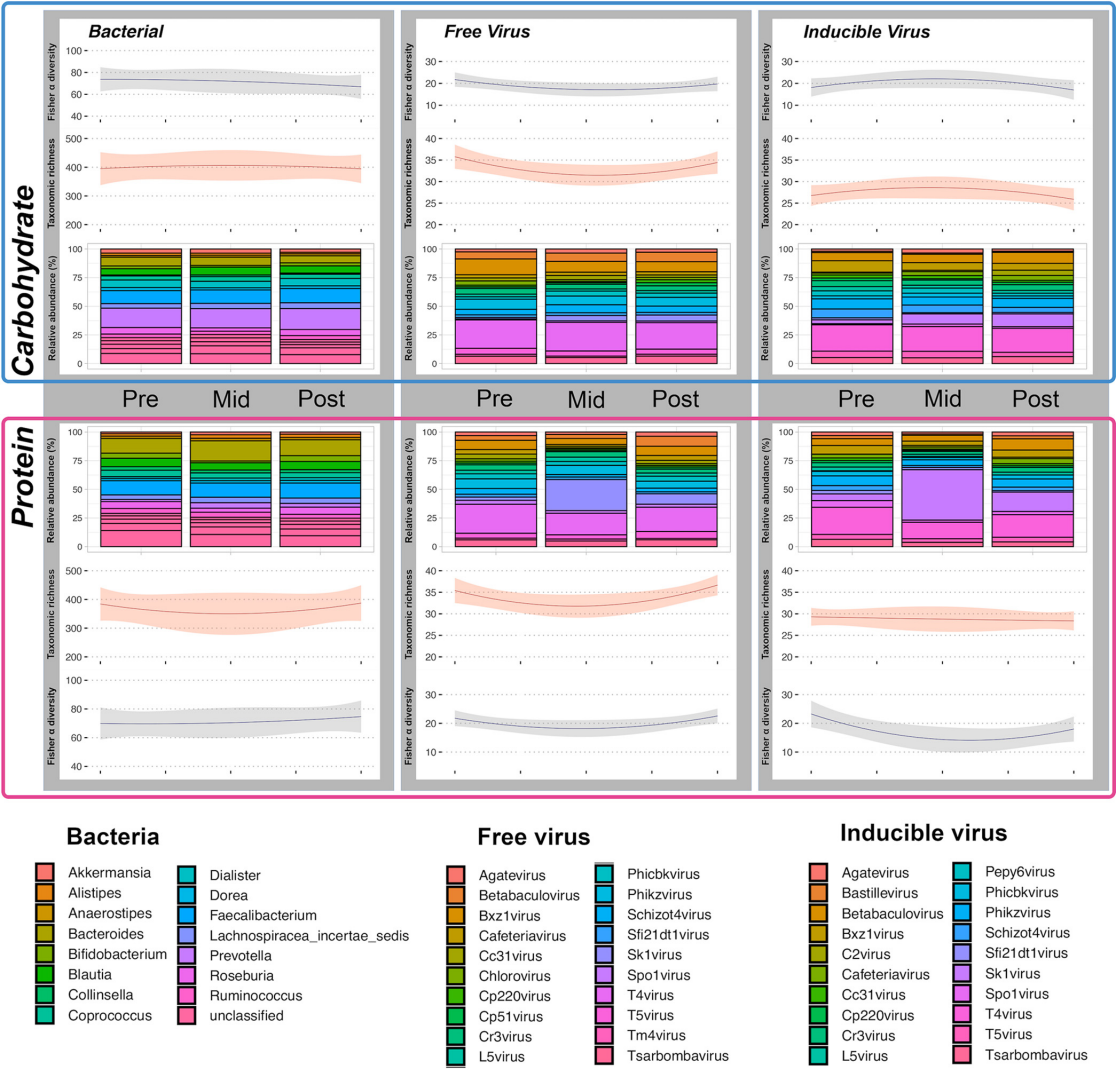
Illustrates overall bacterial, FVP and IV communities measured at pre-, mid-, and postintervention time points for participants enrolled on both the HPD and HPC diets. Longitudinal progression of both Fisher-alpha diversity (navy line), and observed taxonomic richness (red line), are plotted for each community. Shaded area represents 95% confidence interval based on standard error. Microbial taxa included in the bar charts represent the 16 and 20 most abundant bacteria or viruses observed within the data sets, respectively.

Dietary intervention had a significant impact on microbial community composition. Significantly reduced Fisher-alpha diversity of inducible viruses was observed during HPD intervention (KW: *P* = 0.04), with levels failing to return to preintervention levels following cessation (Fig. 2) (Table S1).

10.1128/msystems.00129-22.4 TABLE S1 Alpha diversity of bacterial, FVP, and IV microbial communities was calculated as fisher-alpha index and taxonomic richness. Kruskall-Wallis rank sum test was utilized to identify significant differences between community alpha-diversity of participants on different dietary interventions (HCD versus HPD), and across time points for participants on the same intervention (EARLY versus MID versus LATE). Significant differences were identified between participants on the HCD and HPD diets at preintervention (EARLY) and midintervention (MID) time points, as well as between pre-, mid-, and post- (LATE) time point for patients on the HPD intervention (denoted by an asterisk). Download Table S1, PDF file, 0.03 MB. Copyright © 2022 Furber et al. 2022 Furber et al. https://creativecommons.org/licenses/by/4.0/ This content is distributed under the terms of the Creative Commons Attribution 4.0 International license.

**FIG 3:**
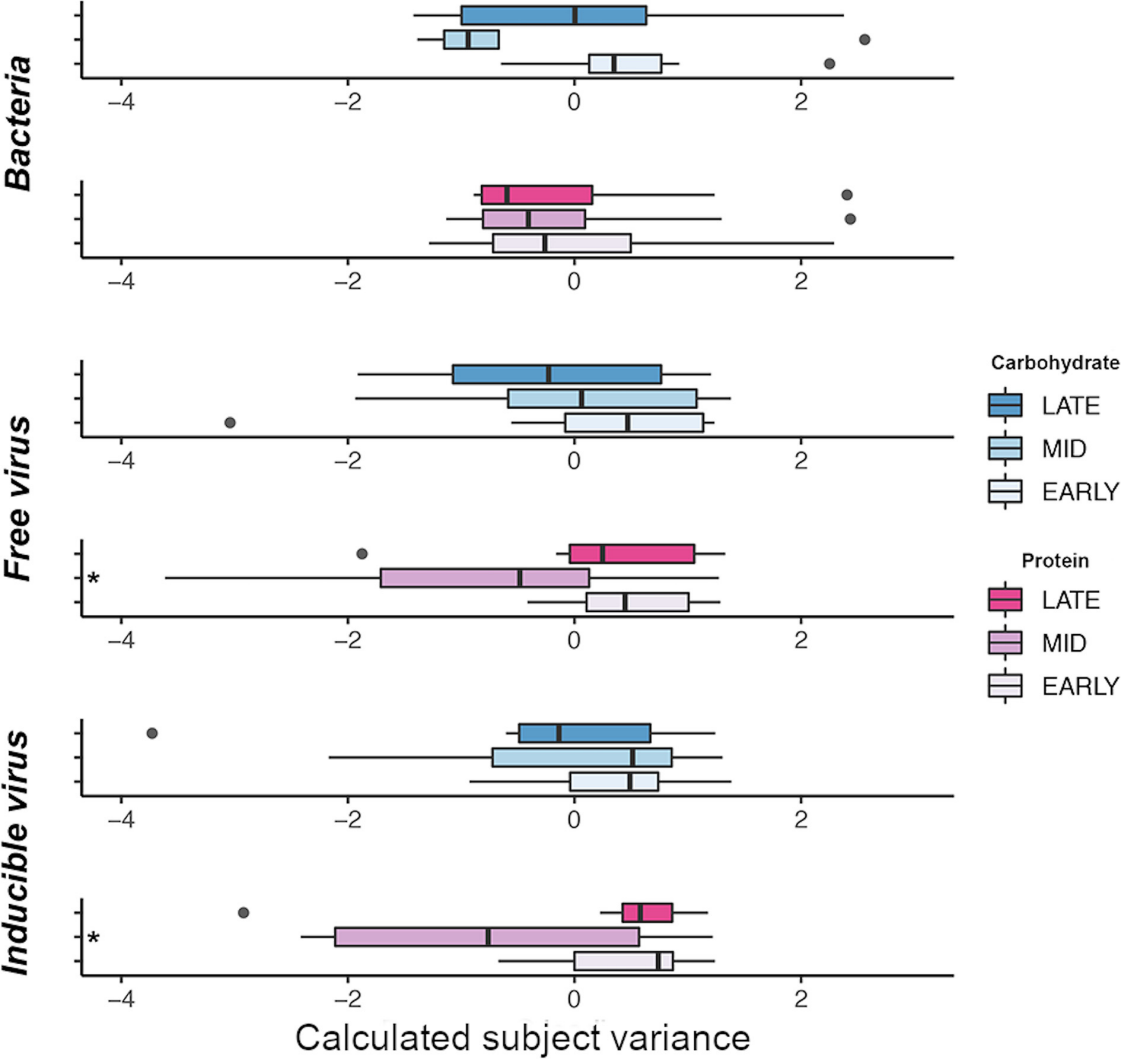
Illustrates shifts in beta-diversity of microbial communities following both HPD (pink), and HCD (blue), interventions. Plotted are the loadings values of the primary component of principle coordinates analysis, time point is depicted by color intensity (post-/late = dark; mid- = light; pre-/early = lightest). The center of each box represents the median, edges extend to upper and lower quantiles and whiskers describe the full range of data, excluding outliers, which are plotted as individual points. Significant shifts in beta-diversity within communities are highlighted by asterisk.

HPDs diet had the greatest impact on viral community composition while HCD diet had the greatest impact on bacterial community composition (Fig. 3). Both free viral particle (FVP) (R^2^ = 0.15; *P* = 0.023), and inducible virus (IV) (R^2^ = 0.16; *P* = 0.016) communities were significantly altered between during HPD intervention (Fig. 3, Table S2). Following cessation of HPD both the FVP and IV communities recovered toward preintervention community compositions (Fig. 3, Fig. S3).

10.1128/msystems.00129-22.6 FIG S3 Shifts in beta-diversity of bacterial, FVP, and IV communities (left) and combined communities, stratified by responder status (right). Data spans pre- (EARLY), mid- (MID), and postintervention (LATE), sampling time points for both HCD (blue) and HPD (pink). Ordinations show the first two principal components of the PCoA computed on Bray-Curtis dissimilarity between samples. Each point represents an individual sample, colored by patient, is shaped by time point and is connected chronologically. The ordinations in the left panel show greater shifts in the viral communities between intervention time points, with communities shifting in a consistent direction during intervention and returning toward the preintervention sample following cessation. Also visible are the highly participant-specific bacterial communities, with samples clustering by participant. The ordinations in the right panel show greater shifts in the microbial communities of unaffected HCD subjects and affected HPD subjects. This is contrasted by tight grouping of samples by subject in the affected HCD and unaffected HPD stratifications. Subjects on the HCD with affected performance performed significantly better during TTE than those unaffected. HPD unaffected subjects performed significantly better during TTE than those unaffected. These results suggest that maintenance of a robust, highly patient-specific gut microbiota is associated with better endurance performance during controlled dietary intervention. Download FIG S3, PDF file, 0.4 MB. Copyright © 2022 Furber et al. 2022 Furber et al. https://creativecommons.org/licenses/by/4.0/ This content is distributed under the terms of the Creative Commons Attribution 4.0 International license.

### Taxonomic features of the microbiota are associated with dietary interventions.

Changes in viral community diversity and composition during HPD intervention is largely contributed to by an expansion in proportional abundance of Sk1virus. This expansion of Sk1virus is visible in both FVP and IV communities (Fig. 2).

**FIG 4:**
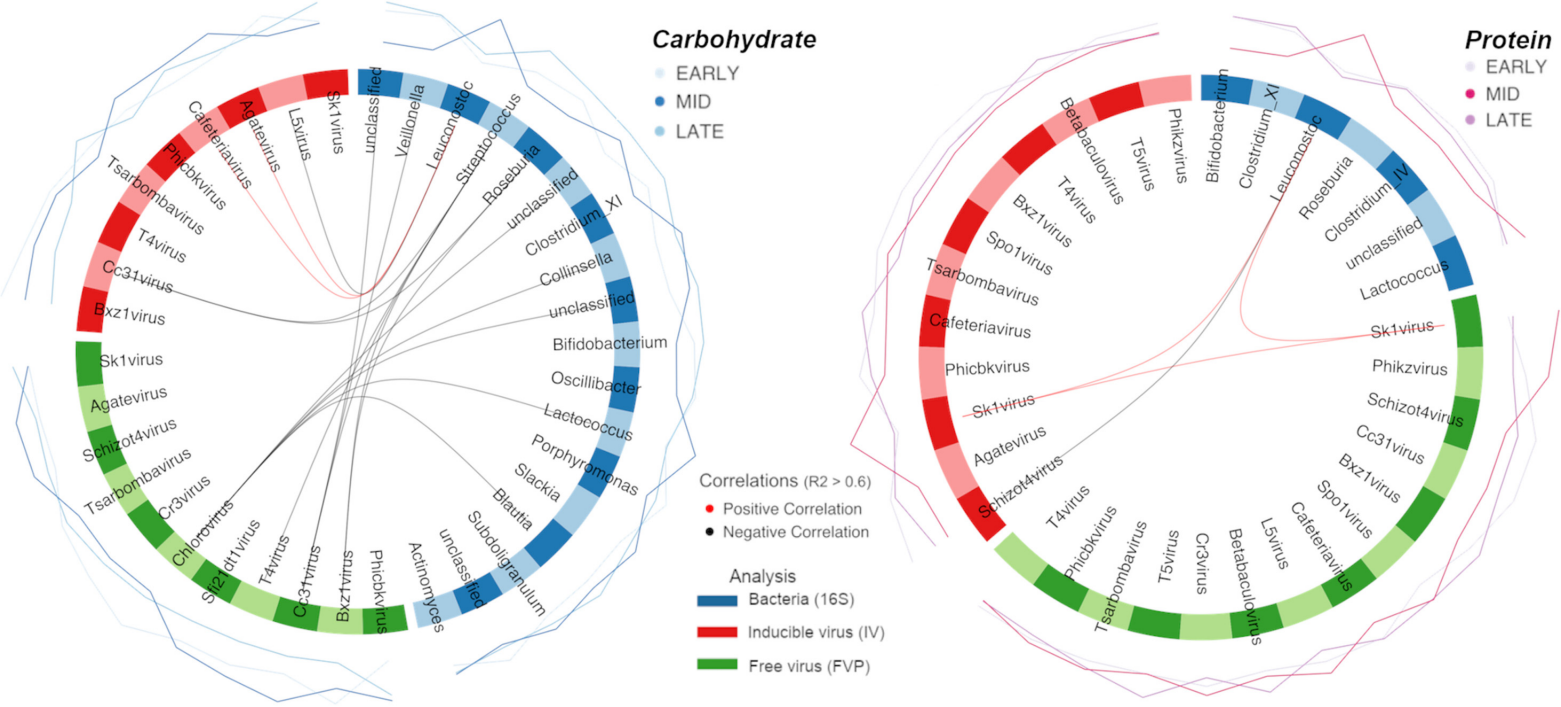
Shows potential biomarkers of both HPD (a) and HCD (b) dietary intervention as identified by sPLS-DA. Significant positive (red), and negative (black), correlations with R^2^ values > 0.6 between features of each community are highlighted by lines joining nodes of bacterial (blue), FVP (green), and IV (red) nodes, forming the outside of the circle. Proportional abundance of each feature is illustrated by external lines colored by time point as in Fig. 2. Effect sizes of each individual feature per community are highlighted in the loadings plot and are colored by the same logic as proportional abundance.

Sparse partial least-squares regression (sPLS-DA), analysis was utilized to identify discriminative features between habitual and HCD or HPD interventions. We combined bacterial, FVP, and IV microbial communities to identify discriminate features between dietary interventions. sPLS-DA highlighted strong positive correlations between proportional abundances of Sk1virus and *Leuconostoc* bacteria, indicating both features are associated with HPD intervention (Fig. 4a). Furthermore, *Leuconostoc* were negatively correlated with IV Schizot4virus, which was reduced during HPD intervention in both viral communities. *Bifidobacterium* spp. proportional abundance was also substantially reduced during HPD intervention.

Despite nonsignificant shifts in the overall community during HCD intervention, proportional abundances of *Leuconostoc* were strongly associated with HCD, as in HPD. *Lactococcus* and *Collinsella* were also both strongly associated with HCD intervention (Fig. 4b). In contrast, Streptococcus exhibited substantially reduced proportional abundance following HCD intervention. Viral features proportionally upregulated in HCD intervention included Cc31virus which was negatively correlated with Streptococcus in both FVP and IV communities, as well as IV *Cafeteria virus*. Conversely, FVP Chlorovirus and IV L5virus were both negatively correlated with *Leuconostoc* and were proportionally reduced during HCD intervention.

Combination of viral and bacterial communities enabled greater discrimination between dietary intervention time points than using any community in isolation as evidenced by sPLS-DA (Fig. S4).

10.1128/msystems.00129-22.7 FIG S4 DIABLO s-PLS-DA ordinations illustrate correlations between principal components of bacterial, FVP (lytic), and IV (temperate), communities for HCD (a) and HPD (b) interventions. Each point represents and individual sample and is colored by time point (pre- = EARLY, mid = MID, post- = LATE). The numbers within the grid layout describe the R^2^ value for each pairwise correlation. Despite the viral communities exhibiting greater discriminative power than the bacterial communities when compared alone, they are highly correlated (see FVP [lytic], and IV [temperate], viruses in the HPD intervention). By combining poorly correlated components, such as the bacterial and viral communities the discriminative power of the model is improved, as illustrated by the ordinations. Download FIG S4, PDF file, 0.1 MB. Copyright © 2022 Furber et al. 2022 Furber et al. https://creativecommons.org/licenses/by/4.0/ This content is distributed under the terms of the Creative Commons Attribution 4.0 International license.

### Gut microbial taxonomic stability is associated with greater performance.

Participants were stratified in to “responders” and “nonresponders” based on percentage change in TTE performance recorded during mid-intervention tests for both HCD and HPD. Based on the results of the linear model (Fig. 1), “responders” were classified as those subjects for which TTE increased more than expected during HCD (*n* = 4) or reduced more than expected during HPD (*n* = 4) intervention. Likewise, four participants were classified as “nonresponders” in both the HPD and HCD interventions.

**FIG 5:**
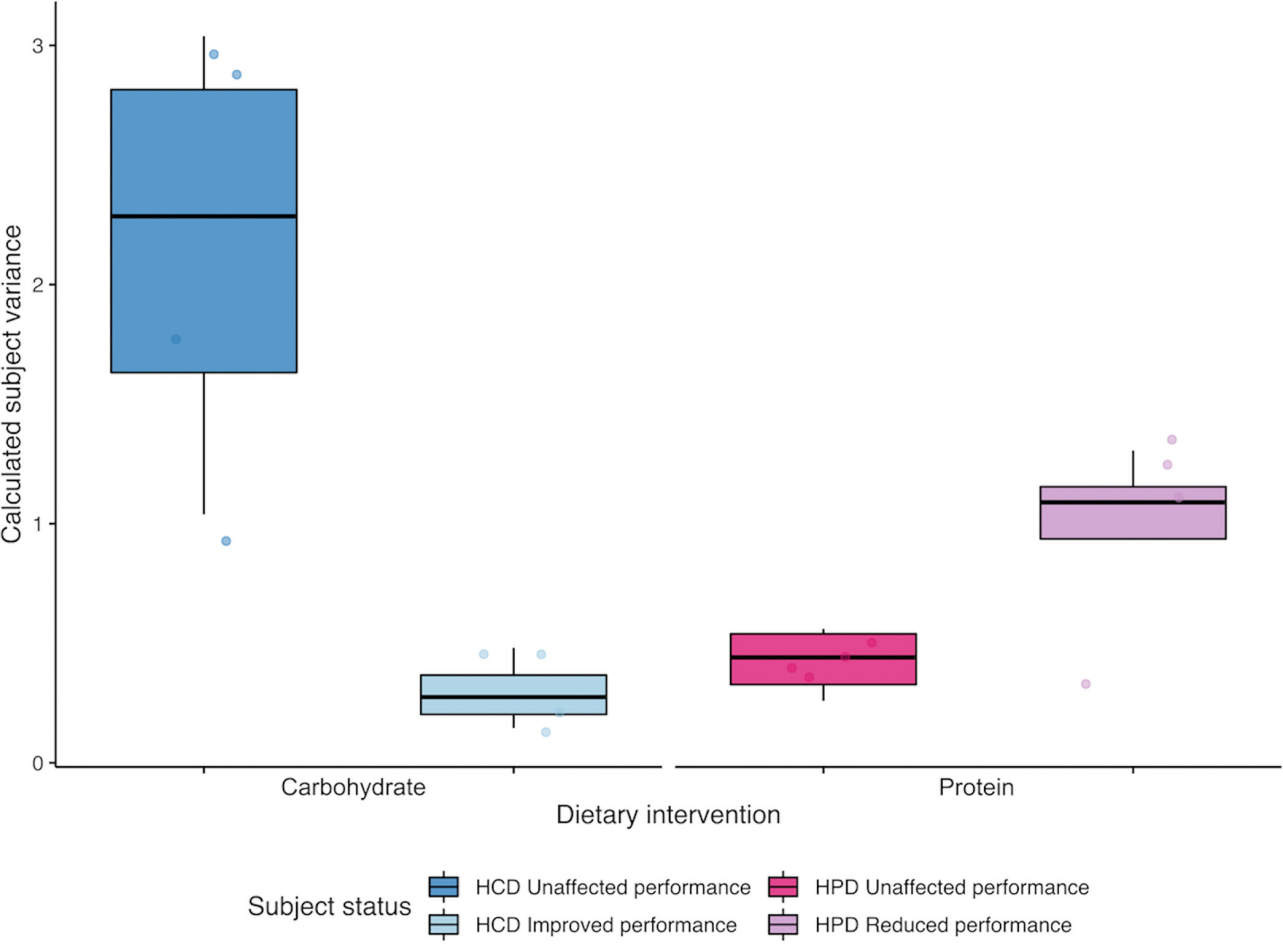
Illustrates within and between subject variance based on Bray-Curtis of combined bacterial, FVP and IV community dissimilarity for participants enrolled in either the HCD (blue), or HPD (pink), interventions. Within subject variance explains the dissimilarity between samples from one individual across all three sampling time points. Between subject variance explains the dissimilarity between samples from different individuals enrolled on the same dietary intervention. Subjects are stratified in to “unaffected” (dark colors), or “improved” and “reduced” groups (light colors), dependent on TTE performance changes within each dietary group. Each point represents variance calculated for an individual subject. Box edges describe the upper and lower quartile ranges while the median is depicted by the central line. Whiskers extend to the full range of data points, excluding outliers.

Combined microbial communities were employed to identify relationships between gut microbial communities and athletic performance during both dietary interventions. Subject variance was used as a measure of microbial stability within individual participants. Microbial communities of improving performers in the HCD intervention were far more stable over time exhibiting lower within-subject community dissimilarity than subjects who did not improve. These observations were coupled with maintenance of a subject-specific gut microbiota in HCD improvers. Nonimproving subjects on the HCD were less distinguishable from one another (Fig. 4, Fig. S3). In contrast, subjects in the HPD with reducing performance exhibited greater microbial turbulence with bigger shifts in gut communities across the study and lower subject-specificity (Fig. 5, Fig. S3). These results suggest that athletic performance may be linked with gut microbial stasis, where athletes harboring stable microbial communities consistently performed best in each dietary intervention compared to those with a more turbulent gut microbiota.

### Assessing gene function in gut microbial communities and inferring impact on athletic performance.

**FIG 6:**
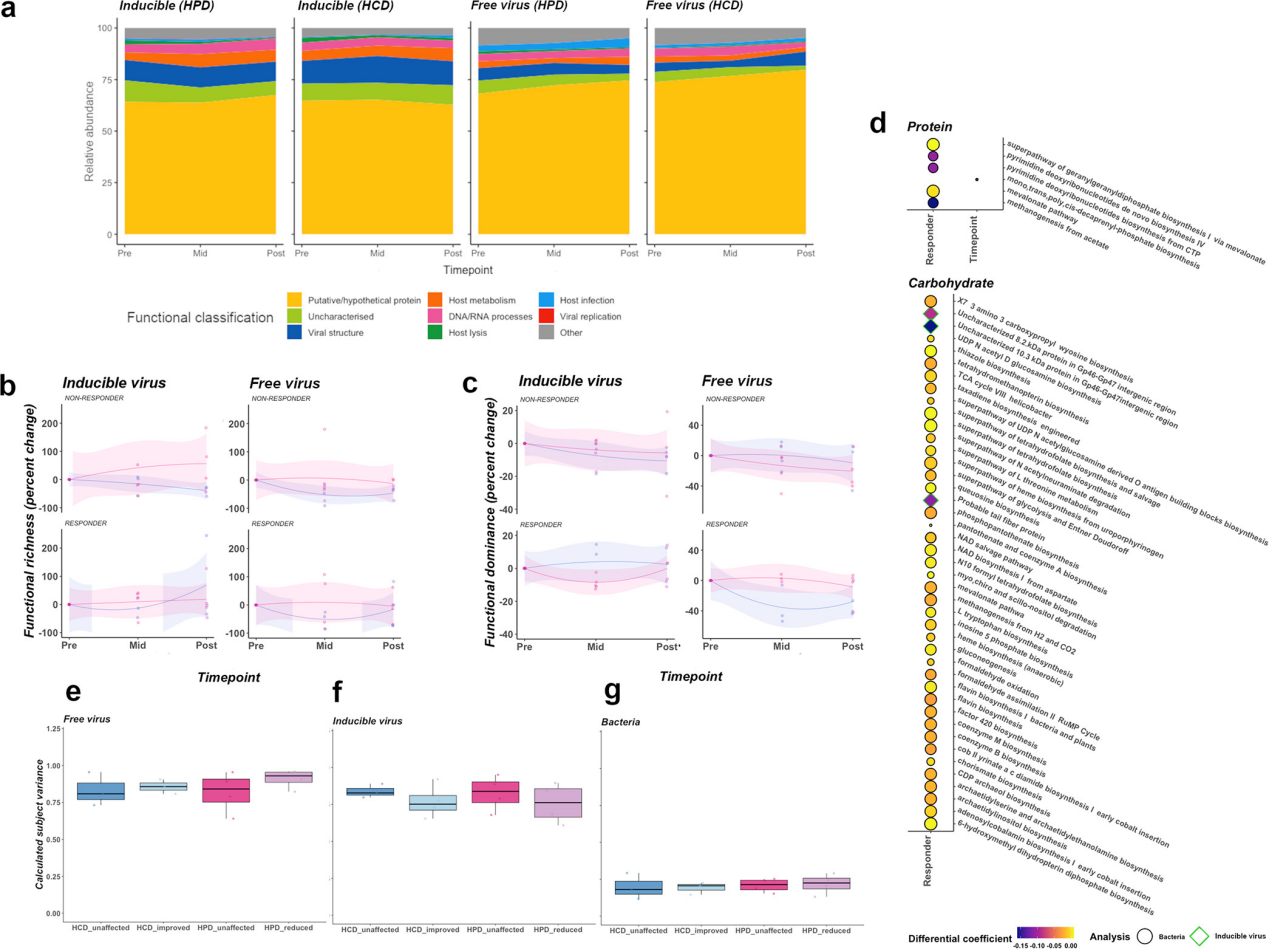
illustrates functional gene compositions of samples. Functional composition across each time point is represented as relative abundance of functional gene classes (a). Changes in functional gene richness (b) and dominance (c) is also presented across sampling time points, stratified by responder status. Each point represents an individual sample and is colored by diet (pink = HPD; blue = HCD). Significantly differential gene features as identified by Maaslin2 are represented by individual bubbles, colored by differential coefficient and stratified by diet (d). Significance (q value) is represented by size of the bubble. Circles represent bacterial gene pathways while diamonds represent viral genes. Associations between microbial functional composition and performance during dietary intervention in FVP (e), IV (f), and bacterial (g) compartments are represented by box and whisker plots, as in Fig. 5.

Better performance during dietary periodization was observed in participants with greater gut microbiota taxonomic stability. We explored whether this was related to functional capacity of the microbiota by interrogating metagenomic data. A total of 12,581 viral orthologous gene (VOG) functional genes were identified across all 48 samples (median 287984, IQR 233105 to 372302). VOG functional genes were grouped into high level classifications as explained in Text S1. Large percentages (up to 75%) of the viral genes identified during this analysis were either hypothetical, putative or unclassifiable by alignment to the VOG database (Fig. 6a). Minor expansions in abundance of temperate viral genes associated with host metabolism were observed during HPD intervention. Genes associated with viral structure were marginally more proportionally abundant during the HCD (Fig. 6a). Percentage change from baseline of functional gene richness and dominance was not different (*P* > 0.05) across either temperate or lytic viral fractions between responders and nonresponders. HCD had a general, though nonsignificant, reducing effect on IV richness (Fig. 6b). Participants who performed best at TTE on the HCD diet, however, recovered from this loss of functional richness whereas nonimprovers continued to suffer reductions in IV functional richness even after intervention cessation (Fig. 6b). Up to a 17% reduction in functional gene dominance was observed in IV-HPD responders and up to 53% reduction in FVP-HCD responders (Fig. 6c), though these differences were not significant. Several significantly differentially abundant pathways (q < 0.25) of bacterial metabolism were observed between responders and nonresponders to both dietary interventions by Maaslin2 analysis (Fig. 6d). Many of these, including inositol degradation, thiazole biosynthesis and NAD salvage/biosynthetic pathways are directly related to carbohydrate utilization. Similarly, to the results of taxonomic analyses, we observed greater change in overall FVP community composition in participants whose performance at TTE was reduced during HPD intervention (Fig. 6e). Additionally, HCD improvers had greater functional community stability in the IV compartment than their nonimproving counterparts (Fig. 6f). Results of functional and taxonomic analyses did not match for FVP HCD or IV HPD cohorts.

10.1128/msystems.00129-22.1 TEXT S1 These provide detailed description of methods employed in the study such as descriptions of inclusion criteria; cohort details; lab visits; assessment of habitual diets and training; intervention diet design and delivery; laboratory methods; bioinformatic processing; and statistical analysis. Download Text S1, PDF file, 0.1 MB. Copyright © 2022 Furber et al. 2022 Furber et al. https://creativecommons.org/licenses/by/4.0/ This content is distributed under the terms of the Creative Commons Attribution 4.0 International license.

## DISCUSSION

This study investigated the influence of two dietary strategies commonly used by athletes on gut microbiome populations and the subsequent impact on running performance. This study is the first to illustrate the impact of short-term, highly controlled HCDs and HPDs on the performance of well-trained endurance athletes. This study showed new data that isocaloric, high-protein diets in highly trained athletes resulted in reduced running performance that was correlated with alterations in gut viral communities. Importantly, changes in the viral communities represent a more sensitive marker of gut stress during dietary intervention than bacterial community analysis alone.

Previous studies have explored changes in the gut microbiota of active versus sedentary individuals finding greater taxonomic (25, 27) and metabolic (26) diversity in active individuals. Additionally, other investigations highlighted the impact of differing levels of dietary protein (24), and carbohydrate (31) on the gut microbiota. These studies focus specifically on gut bacterial communities. However, as highlighted by, Cronin et al., bacteriophage communities can also be implicated in physical performance (32). Here, we utilized a modified approach, stratifying both free and chemically induced lysogenic phages to assess the impact of dietary periodization on each component individually. This current work has identified alterations in viral communities because of dietary change in well-trained athletes. Importantly, this study employed a blinded dietary intervention that was very tightly controlled, and in a highly trained cohort. Consequently, we identified association between the shifts in both bacterial and viral communities in the gut and athletic performance. We were able to discount any variability arising from confounding factors of diet and exercise that exist in previous work by having a well-controlled dietary regimen and homogenously active volunteers. For example, this study cohort harbored distinct gut bacterial communities to that of average human western populations; this was determined by comparison of beta-diversity between stool samples from this cohort and those collected from similar aged males participating in the human microbiome project (Fig. S2) (30). This in turn amplifies the impact of dietary manipulations for even relatively short (7 day) periods.

Of interest to well-trained endurance populations, these data showed that a high-protein diet for 7 days, alongside endurance training, significantly reduced running performance. This manifested as a dramatic −23.3% reduction in mean TTE across participants in the HPD trial arm. Conversely, improved TTE was observed (+6.5%) in athletes on the HCD, which is understandable given the large body of evidence showing high carbohydrate availability is conducive to endurance performance (2, 3). In support of this expired gas analysis performed on this cohort during the 10 km TTE showed there was a greater reliance on fat oxidation following HPD (data previously reported [33]), likely due to lower carbohydrate substrate availability as opposed to the greater use of carbohydrate oxidation. Collectively, these data further illustrate the benefit of HCD for endurance performance in well-trained athletes.

In this study the implementation of a HPD had a greater impact on the viral compartment while HCD had a greater impact on the bacterial microbiota. Increased dietary protein content was associated with reduced IV taxonomic alpha diversity and functional gene dominance, reduced FVP richness, and marked shifts in community compositions of both inducible and free viruses which we hypothesize relates to phage induction, through selective pressure on the bacterial population. Community shifts in both IV and FVP populations were attributable to an expansion in the relative abundance of *Sk1virus* observed in stools. *Sk1viruses* are members of the *Siphoviridae*, which were found to be significantly enriched during HPDs previously (34). Additionally, we found the abundance of *Leuconostoc* bacteria to be positively correlated with *Sk1virus* during HPD intervention. We propose this is a function of a predator-prey expansion relationship as *Sk1virus* are members of the 936-type phage that show broad-range infectivity within the Lactobacillales, of which *Leuconostoc* are a member (35, 36). The co-occurrence of these microbes in individuals consuming HPD may be due to supplementation with proteins derived from whey as these microbes are highly abundant in and associated with fermentation of dairy products (37).

Elevated carbohydrate intake in this study was not associated with the same changes in gut viral communities observed in HPD, but instead had an impact on bacterial community compositions through expansions of *Collinsella* & *Ruminococcus* spp. *Collinsella* have previously been correlated with high levels of circulating insulin (38) and have broad dietary carbohydrate metabolizing potential (39). *Ruminococcus* are well-known degraders of resistant starch in the human gut (31, 40). We propose that the enriched bacterial populations observed during HCD in this study occurred as a direct impact of the increased substrate availability associated with enhanced carbohydrate volumes in the diet. Large shifts in bacterial taxonomy observed in this study correlated with large numbers of differential functional pathways identified in inferred metagenomes. HCD nonresponders failed to recover from loss of IV functional richness following cessation of dietary intervention which may point to links between a lack of functional plasticity and poor performance during HCD intervention. Conversely, the expansion in both inducible and free viral taxonomic communities identified in the HPD may be a result of greater bacterial cell stress in the gut environment due to a lack of fermentable substrate (carbohydrate) in the HPD and highlight the potential negative impacts of HPD.

Analysis of responders and nonresponders to the dietary interventions provide evidence of these proposed differences. For example, responders to the HCD exhibited greater improvements in TTE performance than nonresponders. This improvement was associated with markedly greater taxonomic microbial community stability and marginally greater functional stability of inducible viruses. In contrast, participants whose performance was affected by the HPD performed significantly worse than their peers. This is reflected in the greater change in overall taxonomic communities and FVP functional communities because of the intervention. Maintenance of a subject-specific community and reduced longitudinal variation was observed in athletes who performed better during dietary periodization. Changes in gut communities were associated with lower TTE performance in both dietary interventions. Athletes undergoing dietary periodization with the aim of improving performance would likely benefit from greater gut microbial stability. This is the first time such a phenomenon has been described, and in practical terms may have significant implications for nutrition strategies for training and performance. Constant shifts in dietary intake result in instability of the microbial communities. Athletes that alternate between strict dietary regimes may be more likely to cause microbial instability which may impact performance.

Limitations of this study include the relatively small sample size, lack of mechanistic analysis to link changes in microbial communities more definitively to performance, and the inability to guarantee participant compliance to dietary intervention. However, diets were rigorously controlled and training stimuli were maintained between cohorts, enabling high confidence of trial protocol compliance. This work provides a rare insight into the potential flux experienced in athletes that might engage in dietary periodization and the implications it might have on performance. We performed metagenomic sequencing of viral communities which enabled us to characterize functional genes encoded in the FVP and IV compartments. Unfortunately, the high number of genes reported with unknown function in virus genomes means that databases searches have limited utility. Up to 75% of genes identified in this study aligned to gene targets of unknown or hypothetical function in the VOG database. Future studies should attempt to explore functional characteristics of the viral microbiota, specifically that of bacteriophage, which can have a large impact on bacterial communities in the gut. Furthermore, the functional annotation of bacterial genes performed here was based on inferred metagenomes, predicted from 16S sequencing data. Links between functional plasticity within the gut microbial community, resilience to dietary change and athletic performance should be further explored in larger, metagenomic community analyses. Isolation of gut microbes to characterize carbohydrate and protein substrate utilization of microbial community members, particularly lysogens, would further confirm the hypotheses formed during this study.

### Conclusion.

This study provides new insights into dietary manipulation in athletic populations. Gut microbial stability was associated with greater athletic performance when highly trained individuals underwent dietary periodization. Implementation of an acute high-protein diet resulted in compromised athletic performance in individuals with less stable gut microbiota. We propose this observation is attributable to increased stress within the gut environment. Viral populations provide high sensitivity to determine gut stress and could be used as a marker of microbial instability in future studies. Short-term high carbohydrate diet improved athletic performance and was characterized by subtle alterations to the gut microbiota. Stable microbial communities were associated with better performance. Subjects consistently performed better during physical tests when microbial communities remained relatively unchanged throughout dietary periodization. Athletes, and those supporting them, should be mindful of the potential consequences of altering diet on performance and gut flora and periodize dietary intake (particularly macronutrient distribution) appropriately.

## MATERIALS AND METHODS

### Study design and cohort characteristics.

This study was conducted in accordance with the Declaration of Helsinki (2013) and was approved by the University of Hertfordshire Life and Medical Sciences Ethics Committee (protocol number LMS/PGR/UH/02227). To be considered, participants were required to be “highly trained” endurance athletes and not taking any dietary supplements before or during the trial period (see supplementary materials for detailed inclusion criteria). A minimum of six participants were required per trial arm according to power calculation assessment for sample size (G *power3, Dusseldorf) using α = 0.05; 1 − β = 0.80 based on previous exercise data. Twenty suitable participants were recruited, providing written informed consent (Table 1), with four not completing the study for reasons unrelated to the trial.

**TABLE 1:** Baseline physical characteristics of study participants

Group	Age (yrs)	Height (cm)	Body mass (kg)	Absolute V̇O_2_max (L · min^−1^)	Relative V̇O_2_max (mL·kg^−1^·min^−1^)
HPD (*n* = 8)	25 ± 3.6	179.4 ± 6.4	69.5 ± 3.3	4.38 ± 0.35	63.1 ± 4.8
HCD (*n* = 8)	27 ± 5.0	181.6 ± 3.5	67.6 ± 6.1	4.39 ± 0.28	65.3 ± 6.4

HPD, high-protein diet; HCD, high-carbohydrate diet. No differences were observed in baseline participant characteristics according to allocated starting group (mean ± SD) (*p* > 0.05).

**FIG 7:**
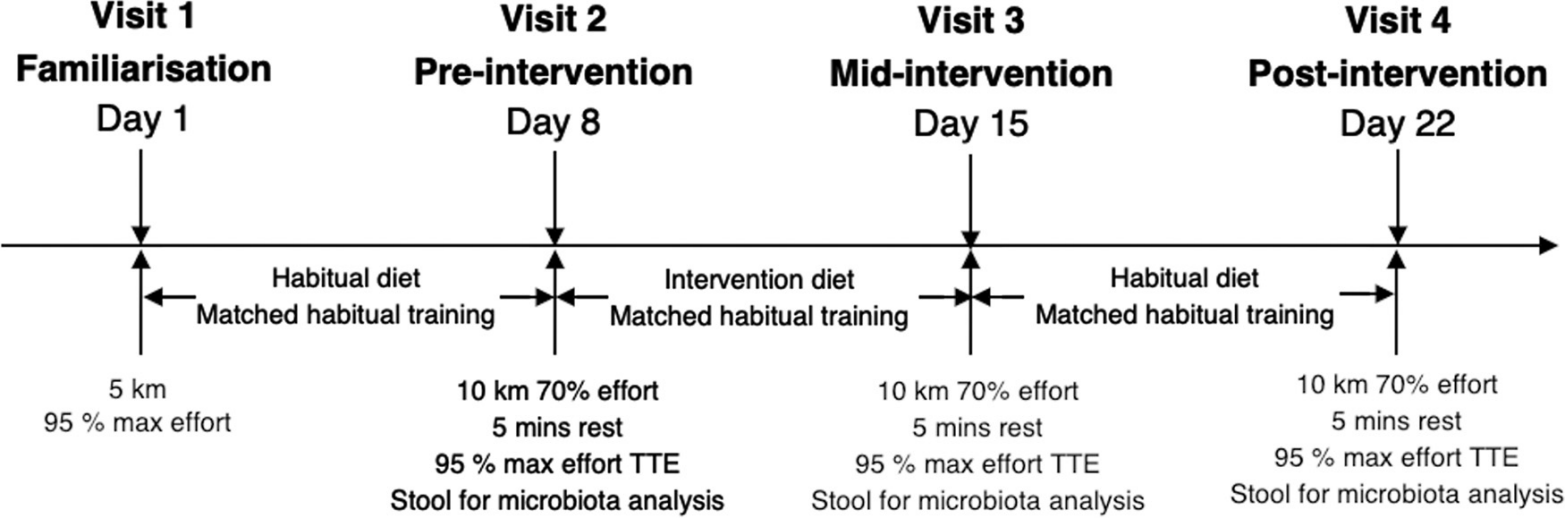
Illustrates the method design employed in this study.

In a double-blind, parallel group, repeated-measures design, participants were randomly assigned to either isocaloric HPD (40% protein, 30% carbohydrate, and 30% fat macronutrient distribution) or energy-matched HCD (10% protein, 60% carbohydrate, and 30% fat macronutrient distribution). While participants remained blinded to which trial arm they were assigned, due to the nature of dietary intervention, we could not blind food items. Further information about diet composition is available in supplementary materials. Participants attended the laboratory on four separate occasions to undertake performance testing and provide samples for microbiota analysis. After a rest day from exercise, no morning physical exertion, and following a minimum 4-h fast, participants arrived at the laboratory via the same mode of transport at the same time each visit. Visits were preceded by 7 days of either habitual or prescribed intervention diet (Fig. 6, and Fig. 7). Participants were requested to maintain training regimes throughout the duration of the study as previously reported (29).

Participants collected their first stool of the day which was transferred to −80°C storage within a maximum of 2 h of passing and remained there until analysis. Participants then completed a 10 km steady state run at 70% V̇O_2_max, had 5 min rest then completed a maximum effort at 95% V̇O_2_max effort until volitional exhaustion (also known as time trial to exhaustion [TTE]). Further details of sample collection and TTE are available in supplementary materials.

### Control of dietary intake.

A 3-day food diary (2 weekdays and 1 weekend day) was used to assess habitual dietary intake prior to visits 2 and 4. During the study briefing participants were instructed how to complete the food dairy and provided with a comprehensive example. The importance of accuracy and detail were emphasized, as was the importance of maintaining current dietary habits. All food diaries were analyzed by the same researcher using dietary analysis software (Nutritics, Dublin, Ireland).

Individual energy intake for the intervention diet was prescribed to match energy expenditure and was calculated using basal metabolic rate (BMR) multiplied by a physical activity factor (PAF) (described in detail in supplementary materials). A total of 34 diet plans (17 HCD, 17 HPD) were formulated by the investigative team, ranging from 1,800 kcal·d^−1^ to 4200 kcal·d^−1^. Participants were assigned the diet closest to their calculated interventional energy intake. An additional 500 kcal·day^−1^ macronutrient-matched meal was provided to ensure participants remained in a positive energy balance during the intervention week. Water was available *ad libitum*, but participants were requested to abstain from drinks containing caffeine or additional energy. The mean dietary intake for both prescribed diets is shown in Table 2.

**TABLE 2:** Calculated, prescribed dietary intake (mean ± S.E.) and macro-nutrient breakdown for each group during the 7-day intervention based on estimated energy requirements

Group	Energy intake		Carbohydrate		Protein		Fat	
	Kcal·d^−1^	Kcal·kg^−1^·d^−1^	g·d^−1^	g·kg^−1^·d^−1^	g·d^−1^	g·kg^−1^·d^−1^	g·d^−1^	g·kg^−1^·d^−1^
HPD	3185 ± 84	48 ± 1.2	239 ± 6.3	3.4 ± 0.9	319 ± 8.4^*a*^	4.6 ± 0.1^*a*^	106 ± 2.8	1.5 ± 0.04
HCD	3281 ± 69	49 ± 1.0	492 ± 10.4^*a*^	7.3 ± 0.1^*a*^	82 ± 1.7	1.2 ± 0.03	109 ± 2.3	1.5 ± 0.03

HPD, high-protein/reduced carbohydrate diet (40/30/30 – protein, carbohydrate, fat ratio); HCD, high carbohydrate/typical protein diet (60/10/30 – carbohydrate, protein, fat ratio).

a Denotes significantly more intake (*p* < 0.05).

### Assessment of training volume.

To ensure any observed changes were due to the intervention diet and not a due to change in training volume, the same weekly training program was followed by each participant throughout the experimental period. Training sessions were logged and submitted online with GPS smartwatches provided to each participant (Garmin Connect, Garmin Ltd., Schaffhausen, Switzerland).

### Isolation of gut microbial DNA.

Stool samples collected during each lab visit were defrosted and homogenized in 3 mL of ice cold, sterile 1x Phosphate buffered saline (PBS) then allowed to settle for 5 min. Also, 1 mL supernatant was sub-sampled for viral community analysis. The viral sub-sample was processed as per modified protocols previously described (41). Briefly, the sub-sample was centrifuged at 4,000 rpm and 4°C for 10 min. The supernatant was used for free viral particle (FVP) analysis and the pellet used for inducible virus (IV) community analysis. The IV pellet was resuspended in 1 mL sterile 1x PBS and incubated with norfloxacin at 1 μg/mL for 1 h at 37°C. The incubated pellet was then centrifuged at 4,000 rpm and 4°C for a further 10 min and the supernatant taken for DNA isolation.

Prior to isolation of FVP, IV, and bacterial and fungal DNA, all samples were treated with of 1 μL TURBO DNase and 1 μL RNase Cocktail (Life Technologies, CA, USA) to deplete any free DNA in solution. Treatment consisted of 30 min at 37°C before inactivation at 65°C for 1 min in 15 mM EDTA. Viral DNA was extracted as per manufacturer’s instructions using NORGEN Phage DNA Isolation Kits (Geneflow Limited, Lichfield, UK). Bacterial and fungal DNA was isolated from the remaining homogenized stool following removal and processing of the 1 mL viral supernatant using DNEasy PowerLyzer PowerSoil DNA Isolation kits (Qiagen, DE), as per manufacturer’s instructions.

### Sequencing and processing of gut microbial DNA.

All sequencing was performed by NUOmics (Newcastle, UK). Viral DNA metagenome sequencing libraries were processed via the Illumina Nextera XT prep (Illumina, Saffron Waldon, UK). Libraries were sequenced on the Illumina MiSeq using V3 2 × 300 bp chemistry. Viral sequence reads were trimmed and quality filtered using *Trimmomatic* (42) and *Sickle* (43). Sequences homologous to the human genome were culled using *KneadData* in the *bioBakery* environment (44). Taxonomic assignment was achieved by aligning sequences against the NCBI nr database using a kmer size of 20% and 70% identity threshold with *blastn* (45). Lowest common ancestor and relative abundances were calculated in *MEGAN6* (46). Functional annotation of viral sequences was performed by aligning sequences to those in the VOG database (https://vogdb.org, accessed 6/12/21) using default *VIBRANT* (47) parameters. Normalized abundance of identified VOGs per sample was calculated using *bwa mem* (48), *samtools* (49), *bamtools* (50), and *bedtools* (51). To discriminate between multiple target alignments of a single sequence against the VOG database, a hierarchical preference criterion was applied. Firstly, actual classifications were preferred over putative ones. If multiple actual classifications were present for a single sequence, greater *VIBRANT* outputted *V.score* was preferred, followed by lower *E.score*. Custom R scripts merged feature counts per sample to create feature tables for statistical analysis.

Bacterial and fungal DNA amplicon sequencing libraries were prepared as per the Schloss protocol (52). Bacterial amplicons targeted the V4 region of the 16S rRNA gene while fungal amplicons targeted the ITS1 and ITS2 regions of rRNA spacer genes. Both bacterial and fungal libraries were sequenced on the Illumina MiSeq using V2 2 × 250 bp chemistry. Taxonomic assignment of bacterial and fungal sequence reads was performed in Mothur (53). Briefly, paired end reads were trimmed, merged, quality filtered, and clustered into *de novo* operational taxonomic units (OTUs) using the OptiClust method (54). OTUs were aligned to SILVA (bacterial [55]), and UNITE (fungal [56]), databases to determine taxonomy. *PiCrust2* (57) was used to infer functional metagenomes from, chimera filtered, 97% homology bacterial OTU reference sequences classified using “ready-to-wear” classifiers specific for human stool communities (58, 59) by aligning to the Greengenes (13_8) database (60) in *QIIME2* (61).

All raw sequencing data is freely available at European Nucleotide Archive (ENA) under study accession PRJEB45703.

### Statistical analysis.

Statistical analyses were performed using R (62). Normality of data was verified by the Shapiro–Wilk test. Kruskal-Wallis rank-sum test (KW) was used to compare means of continuous data. Where appropriate, a Bonferroni *post hoc* test was used.

Alpha diversity of microbial communities was assessed by richness, diversity (Fisher-alpha), and dominance (Simpson) metrics while beta diversity was assessed by Bray-Curtis dissimilarity for taxonomic analyses and Canberra distance for functional gene composition analyses in the *vegan* package (63). All taxonomic diversity metrics were calculated at the genus level. Adonis PERMANOVA was used to identify metadata variables significantly impacting community compositions utilizing the “*strata*” function to account for repeated measures. Microbial stability/change was assessed using calculated subject variance, being the average range of loadings values from the primary principal component of dissimilarity between microbial communities from a single trial participant over time. Discriminatory features of combined bacterial and viral microbiota between intervention time points were identified by sparse partial least-squares regression based on combined bacterial, FVP and IV communities. The model was tuned to prune noninformative variables using the DIABLO method (64). Significantly differential features within functional gene analyses were identified using *Maaslin2* (65). *Phyloseq* (66), *mixOmics* (67) and *ggplot2* (33) packages were used to visualize data. STORM checklist: https://zenodo.org/record/6530945#.YnjV0FzMLAg.

10.1128/msystems.00129-22.5 TABLE S2 Beta-diversity of bacterial, FVP, and IV communities was calculated as Bray-Curtis dissimilarity. Adonis PERMANOVA was utilized, accounting for repeated measures using the “strata” function to identify significant differences between community beta-diversity of participants across the intervention time points and between individual patients. Individual patient had the greatest impact on community beta-diversity, explaining 80%, 51%, and 52% of dissimilarity for bacterial, FVP, and IV communities, respectively. Further significant differences between community beta-diversity was observed between time points within the HPD dietary intervention for the FVP and IV communities. To further identify the time point at which viral communities were significantly different within dietary interventions pairwise Adonis PERMANOVA was utilized, including participant as a repeated measure. Significant dissimilarity in both FVP and IV communities were observed between preintervention (EARLY) and midintervention (MID) time points, indicating HPD altered the gut viral community during intervention however, postintervention the viral community began to recover towards the preintervention composition. Significant values are highlighted with an asterisk. Download Table S2, PDF file, 0.05 MB. Copyright © 2022 Furber et al. 2022 Furber et al. https://creativecommons.org/licenses/by/4.0/ This content is distributed under the terms of the Creative Commons Attribution 4.0 International license.
