# Supplementary material for: Gut Microbial Stability is Associated with Greater Endurance Performance in Athletes Undertaking Dietary Periodization
Source: mSystems. 2022 May 17;7(3):e00129-22. doi: 10.1128/msystems.00129-22 (PMC9238380; doi:10.1128/msystems.00129-22)

Supplementary figure 1 - Library sizes per gut microbial community sample

Dynamic changes in the gut microbiota in response to acute high protein and high carbohydrate diets in endurance athletes.

Furber, M.J.W., Young, G.R., Holt, G., Pyle, S. Howatson, G., Roberts, M.G., Roberts, J.D. and Smith, D.L

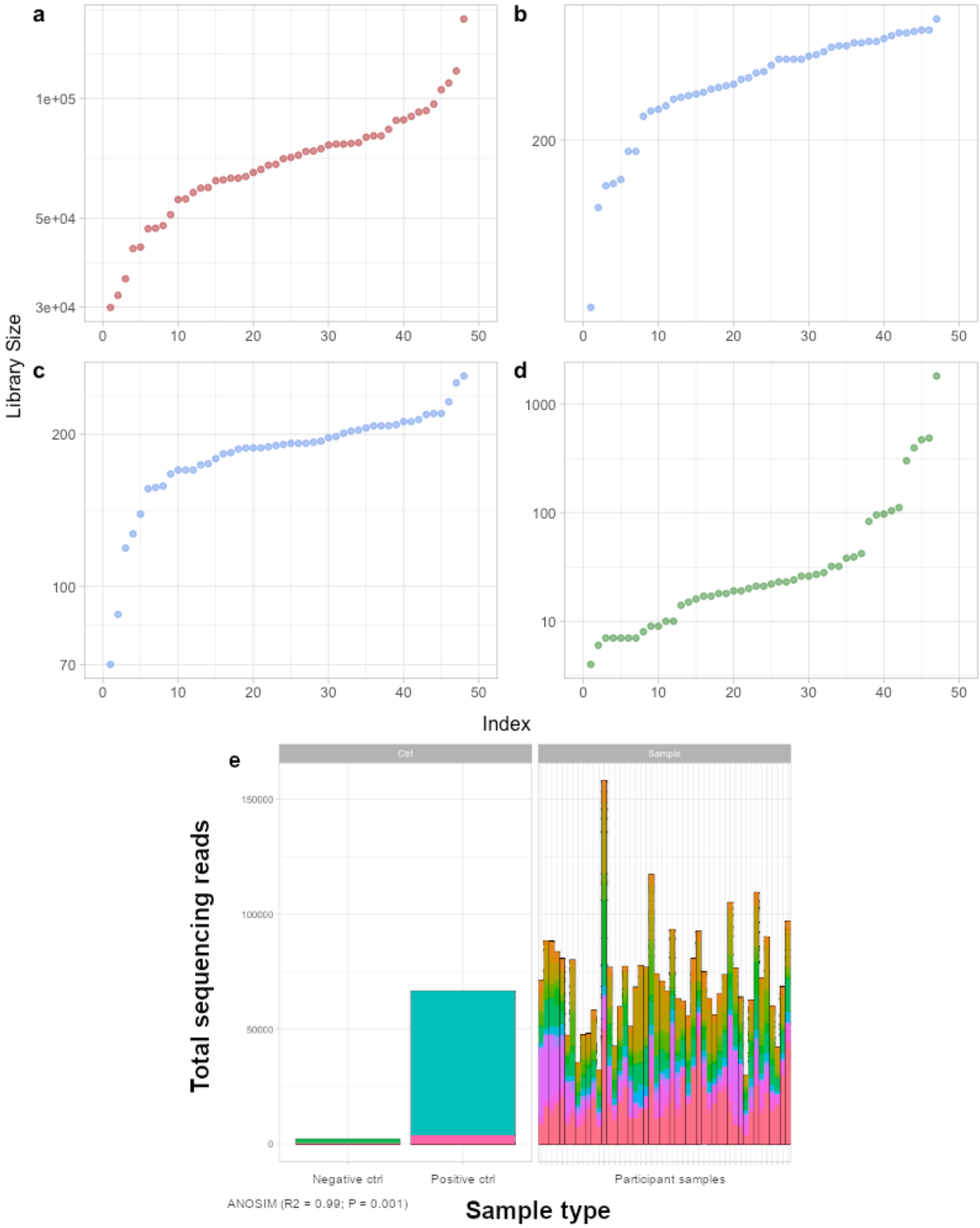

Supplement: FIG S1 [file msystems.00129-22-s0002.pdf]
