# Supplementary material for: Gut Microbial Stability is Associated with Greater Endurance Performance in Athletes Undertaking Dietary Periodization
Source: mSystems. 2022 May 17;7(3):e00129-22. doi: 10.1128/msystems.00129-22 (PMC9238380; doi:10.1128/msystems.00129-22)

Supplementary figure 2 – Beta-diversity within bacterial communities in this study is significantly lower than within bacterial communities in the Human Microbiome Project

Dynamic changes in the gut microbiota in response to acute high protein and high carbohydrate diets in endurance athletes.

Furber, M.J.W., Young, G.R., Holt, G., Pyle, S. Howatson, G., Roberts, M.G., Roberts, J.D. and Smith, D.L

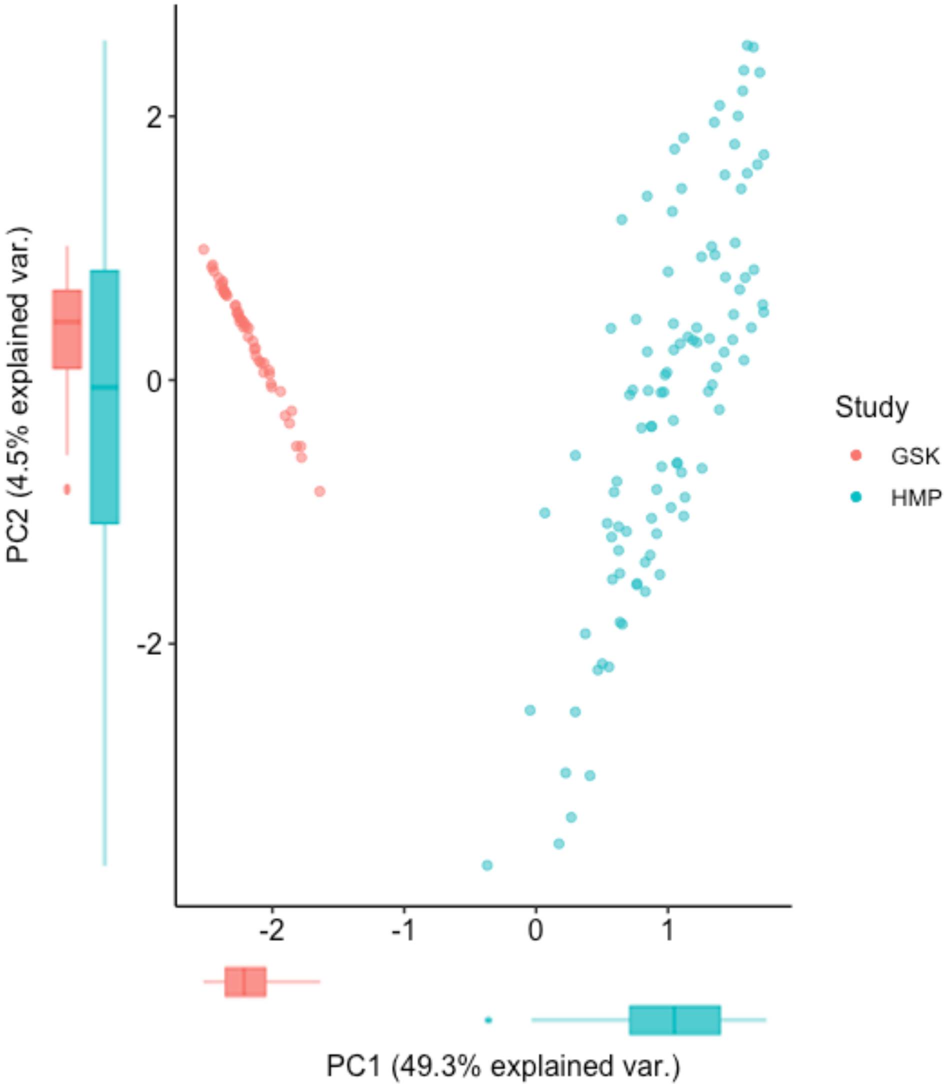

Supplement: FIG S2 [file msystems.00129-22-s0003.pdf]
