# Supplementary material for: Gut Microbial Stability is Associated with Greater Endurance Performance in Athletes Undertaking Dietary Periodization
Source: mSystems. 2022 May 17;7(3):e00129-22. doi: 10.1128/msystems.00129-22 (PMC9238380; doi:10.1128/msystems.00129-22)
Supplement: TABLE S1 [file msystems.00129-22-s0006.pdf]

Supplementary table 1 – Adonis PERMANOVA results: gut microbial alpha-diversity
Dynamic changes in the gut microbiota in response to acute high protein and high carbohydrate diets in endurance athletes.

Furber, M.J.W., Young, G.R., Holt, G., Pyle, S. Howatson, G., Roberts, M.G., Roberts, J.D. and Smith, D.L

	BACTERIAL	FVP	IV
**Fisher-alpha**			
HCD vs HPD (early)	0.34	0.60	0.04*
HCD vs HPD (mid)	0.67	0.46	0.04*
HCD vs HPD (late)	0.52	0.25	0.30
EARLY vs MID vs LATE (HCD)	0.54	0.64	0.15
EARLY vs MID vs LATE (HPD)	0.85	0.31	0.04*
**Richness**			
HCD vs HPD (early)	0.34	0.40	0.21
HCD vs HPD (mid)	0.60	0.60	0.07
HCD vs HPD (late)	0.60	0.25	0.12
EARLY vs MID vs LATE (HCD)	0.56	0.28	0.34
EARLY vs MID vs LATE (HPD)	0.83	0.13	0.18
