# Supplementary material for: Gut Microbial Stability is Associated with Greater Endurance Performance in Athletes Undertaking Dietary Periodization
Source: mSystems. 2022 May 17;7(3):e00129-22. doi: 10.1128/msystems.00129-22 (PMC9238380; doi:10.1128/msystems.00129-22)

<u>Supplementary figure 3 – Shifts in beta-diversity of gut microbiota are visible during dietary intervention and are associated with endurance performance</u>

<u>Dynamic changes in the gut microbiota in response to acute high protein and high carbohydrate diets in endurance athletes.</u>

Furber, M.J.W., Young, G.R., Holt, G., Pyle, S. Howatson, G., Roberts, M.G., Roberts, J.D. and Smith, D.L

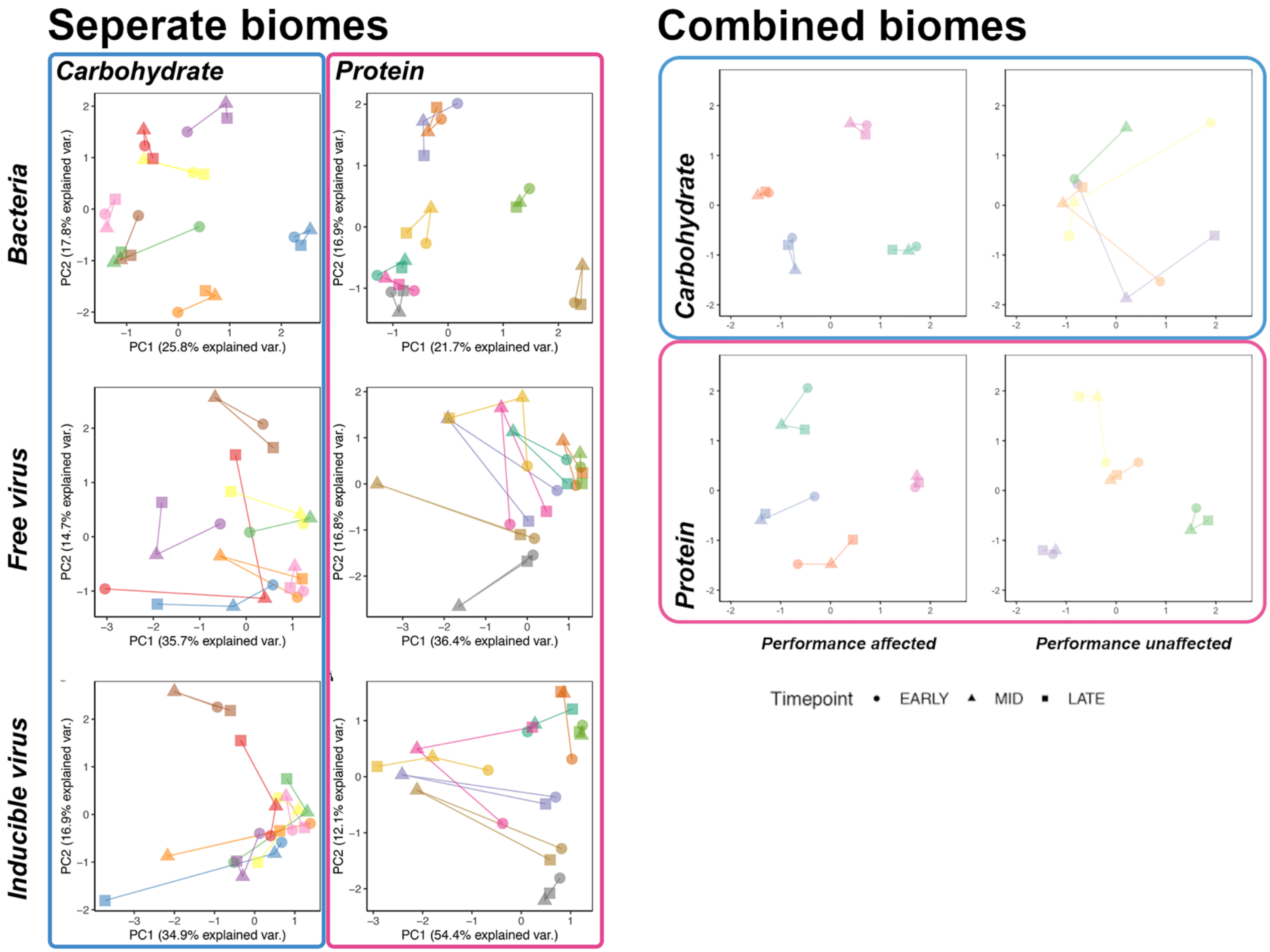

Supplement: FIG S3 [file msystems.00129-22-s0004.pdf]
