# Supplementary material for: Gut Microbial Stability is Associated with Greater Endurance Performance in Athletes Undertaking Dietary Periodization
Source: mSystems. 2022 May 17;7(3):e00129-22. doi: 10.1128/msystems.00129-22 (PMC9238380; doi:10.1128/msystems.00129-22)

Supplementary figure 4 - Combination of viral and bacterial communities enables greater discrimination between dietary time points

Dynamic changes in the gut microbiota in response to acute high protein and high carbohydrate diets in endurance athletes.

Furber, M.J.W., Young, G.R., Holt, G., Pyle, S. Howatson, G., Roberts, M.G., Roberts, J.D. and Smith, D.L

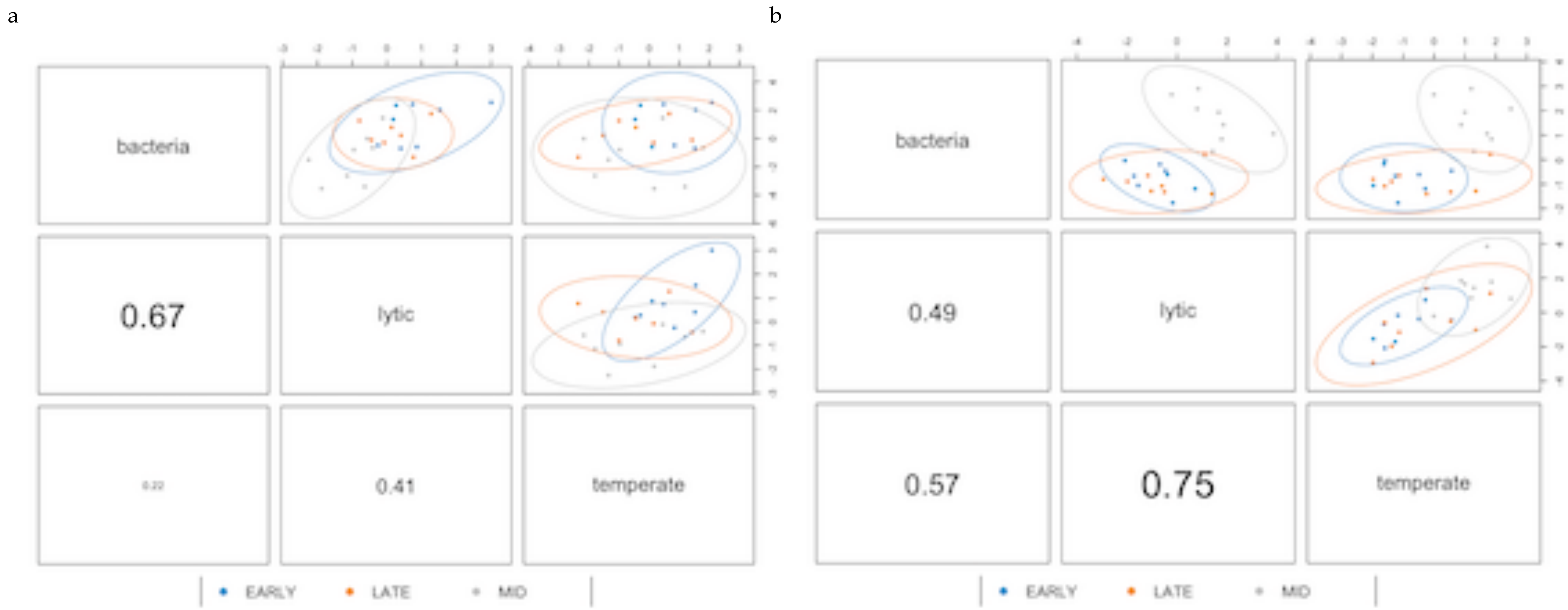

Supplement: FIG S4 [file msystems.00129-22-s0005.pdf]
