# Supplementary material for: Gut Microbial Stability is Associated with Greater Endurance Performance in Athletes Undertaking Dietary Periodization
Source: mSystems. 2022 May 17;7(3):e00129-22. doi: 10.1128/msystems.00129-22 (PMC9238380; doi:10.1128/msystems.00129-22)
Supplement: TEXT S1 [file msystems.00129-22-s0001.pdf]

Supplementary methods

Dynamic changes in the gut microbiota in response to acute high protein and high carbohydrate diets in endurance athletes.

Furber, M.J.W., Young, G.R., Holt, G.S, Pyle, S. Howatson, G., Roberts, M.G., Roberts, J.D. and Smith, D.L

Inclusion criteria

Participants had to be highly trained, male endurance athletes. Highly trained was defined as completion of 10 km <38 minutes or 5 km <18 minutes in the previous 12 months), have a running history > 2 years, training ≥ 4 times per week with no significant change in training load or body mass in the previous 3 months. Further inclusion criteria required participants to be absent of any underlying medical conditions, otherwise apparently healthy and not following a restrictive dietary regime or suffer any known food allergies or intolerances.

Trial completion cohort

Of the 20 highly trained male endurance athletes entering the trial, three participants withdrew due to injury or illness (unrelated to the trial) and one additional volunteer was unable to start the second trial after the HPD intervention (reasons unrelated to the trial).

Lab visits

Visit 1 consisted of briefing, familiarisation and $\dot{V}O_{2}max$ testing. The briefing included clear verbal and diagrammatic guidance on how to collect stool samples using Fe-Col® Faecal Sample Collection kit (Alpha Laboratories, Hampshire, UK) prior to each visit.

Participants then followed their habitual dietary intake for 7 days prior to completing pre-intervention performance testing at visit 2. Intervention diets (HPD or HCD) were then supplied, and participants were informed to follow these for 7 days prior to completing mid-intervention performance testing during visit 3. In the 7 days before the fourth and final lab visit participants returned to their habitual dietary intake prior to a final, post-intervention performance test.

Participants were advised to arrive at the laboratory using the same mode of transport and requested to refrain from morning physical exertion. Stool samples were collected either at home or in the laboratory from the first stool in the morning of each testing visit using Fe-Col® Faecal Sample Collection kit (Alpha Laboratories, Hampshire, UK) according to previous instruction. Samples were delivered to the investigator at room temperature then immediately placed in a -80 freezer and stored at that temperature until analysis. Participants then completed a 10 km steady state run at 70% $\dot{V}O_{2max}$, had 5 minutes rest then completed a maximum effort at 95% v $\dot{V}O_{2max}$ max effort until volitional exhaustion.

Habitual Dietary Intake.

A 3-day food diary using estimated household measures was used to collect habitual dietary intake prior to the first and third testing sessions. During the study briefing the participants were instructed how to complete the food dairy and was provided with a comprehensive example. The importance of accuracy and detail was emphasised (e.g., item weight, portion size, meal breakdown, fluid intake), as was the importance of maintaining current dietary habits and documenting all food and drink consumed. Consumption of dietary supplements was considered a contraindication to participation in the trial; thus, all participants were free from dietary supplements in the period preceding and during the trial protocol. Participants were requested to record dietary intake on 2 weekdays and 1 weekend day each week between visit 1 and visit 2 and repeat again between visit 3 and visit 4. All food diaries were analysed by the same qualified nutritionist using Nutritics (Nutritics, Dublin, Ireland).

Assessment of Training Volume

It was important that any observed changes were due to the intervention diet and not a due to change in training volume. Therefore, training volume was controlled throughout the experimental period.

Participants were requested to maintain the same weekly training programme throughout the study and were requested to replicate, as closely as possible, each training session on the same day and time each week, logging all training sessions with the GPS watch provided. The training sessions were automatically uploaded online (Garmin Connect, Garmin Ltd, Schaffhausen, Switzerland) for the investigative team.

Intervention dietary prescription

Individual calorie intake for the intervention diet was calculated using basal metabolic rate (BMR) multiplied by a physical activity factor (PAF). The equation of Henry (2005) was used to calculate BMR. PAF was calculated depended on occupational and exercise level using a modified Nutritics equation (Harris & Benedict, 1918):

PAL = (0.66 x occupational factor) + (0.33 x exercise factor)

Unless training twice a day the participant was deemed to be 'Very' active, if training twice a day 'Extra' active was selected. Occupational multiplication factor was selected through a series of work-related questions.

A total of 34 (17 CHO, 17 PRO) diet plans were prescribed by the investigative team and created by Soulmatefood® (Waterfoot, Lancashire, UK). Starting at 1,800 kcal the diets increased by 150 kcal for each. Participants were assigned the diet closest to their calculated interventional energy intake. The greatest difference between estimated intervention diet and prescribed diet was 75 kcal per day. All intervention meals were delivered to the participant's door, each receiving two deliveries; the first delivery contained three day's food, the second four day's food. Each day food consisted of 5 pre-packaged/cooked meals (breakfast, morning snack, lunch, afternoon snack and dinner). Matching energy intake to energy expenditure is challenging and it was important that the participants were not in a negative energy balance for this study therefore in addition to the calculated dietary intake, a further 500 kcal•day^{-1} macronutrient-matched meal was provided. Included with the delivery was a daily menu with consumption instructions. Participants were briefed that the additional meal was only to be consumed if still hungry after consuming the standard diet and were required to document if this additional meal was consumed. Participants were requested to remain hydrated through the day, only consume food provided and to only drink water or drinks free from caffeine or additional energy.

Table 4. Calculated mean dietary intake and macro nutrient breakdown for each group during the 7-day intervention.

Group	Dietary intake (kcal•day^{-1})	CHO (•day^{-1})		PRO (•day^{-1})		FAT (•day^{-1})	
		g	g•kg^{-1}	g	g•kg^{-1}	g	g•kg^{-1}
PRO	3185 ± 237	239 ± 18	3.4 ± 0.3	319 ± 24	4.6 ± 0.3	106 ± 8	1.5 ± 0.1
CHO	3281 ± 195	492 ± 29	7.3 ± 0.4	82 ± 5	1.2 ± 0.1	109 ± 7	1.5 ± 0.1

Prescribed dietary intake (kcal•day^{-1}, mean ± SD) and macronutrient breakdown for the prescribed intervention diets for both groups.

10 km steady state treadmill run

Following a resting 20 µL capillary blood sample, participants completed a standardised warm-up consisting of a 5 minutes jog at 10 km•h-1 on the treadmill, followed by self- directed stretching. A facemask (attached to the online gas analyser) was fitted and stayed on throughout the trial. Participants were asked to straddle the treadmill and the belt speed was set to the intensity representative of 75% VO2max (as calculated from the familiarisation session). Once the treadmill belt was up to speed whilst holding on to the treadmill arms, the participant lowered stepped onto the belt and began running. As soon as the participants let go of the treadmill arms the time was noted and the sub-maximal exercise trial started. Participants had minimal visual or audible stimulus throughout the trial. After each kilometre was

completed the same words were repeated to the participant: Well done that is another kilometre completed, 'X' to go, a capillary blood sample and rate of perceived exertion (RPE) (BORG 1070) were then collected. Participants were also informed of the distance completed at 9,500 meters. At 9,850 meters the final capillary blood sample and RPE were recorded. At 10,000 meters the participant held onto the arms of the treadmill and stepped off. A stopwatch was started immediately to time 5-minute recovery.

Time to Exhaustion: 95% maximal sustainable effort (MaxSE).

The treadmill belt was set to a speed equivalent to 95% vVO_2max (as calculated from the familiarisation session), participants straddled the belt and the facemask was fitted again. Once the 5 minutes recovery was up participants supported their weight on the arms of the treadmill and started running on the belt. Once comfortable the participants let go and the stopwatch was started to record the duration of the effort. No visual or audible encouragement was permitted throughout the trial. Participants ran until volitional fatigue, once reached participants straddled the treadmill. The stopwatch was stopped as soon as the participant reached out for the treadmill arms to support their weight. Immediately upon volitional fatigue a final capillary blood sample was collected. The same investigator timed all MaxSE efforts throughout the study.

Sample isolation and DNA extraction

All fecal samples were stored at – 80 º C, and all extractions were carried out on ice where appropriate. Kit negatives were processed with each batch. All DNA samples were stored at -80 °C.

Free Viral particle (FVP) isolation: 120 mg of stool was homogenised in 3 mL of ice cold, sterile 1 x PBS and allowed to settle for 5 min. 1ml of the supernatant was used for viral DNA extraction. This was centrifuged at 4000 rpm at 4 ° C for 10 minutes, and the supernatant removed. The pellet was used for the inducible virus protocol.

Induced viral particle isolation: The pellet from 'Free viral particle isolation' was resuspended in 1 mL sterile, room temperature, 1 x PBS. Virus induction was processed with addition of norfloxacin at 1 µg/mL and incubated for 1 hour at 37 ° C. After incubation each sample was centrifuged at 4000 rpm at 4 ° C for 10 minutes. 1 ml of the supernatant was used for viral DNA extraction.

Viral DNA extraction

Prior to DNA extraction, non-encapsulated DNA was depleted through 1 x round of 1 µL of TURBO DNAse and 1 µL of RNAse Cocktail (Life Technologies Limited), the solution was incubated at 37 °C for 30 min. The DNAse and RNAse were inactivated using heat at 65 °C and 15 mM EDTA final concentration for 10 min. DNA was extracted from the free and chemically induced viruses using the NORGEN Phage DNA Isolation Kits (Geneflow Limited, Lichfield, UK). The manufacturers protocol was modified as per Tariq *et al*. (2015), for removal of bacterial/eukaryotic chromosomal DNA. Kit negatives were processed with each batch.

Total DNA isolation for microbial community analysis

DNA was extracted from 1 mL of homogenised stool, harvested as a pellet after centrifugation at 4000 rpm at 4º C for 10 minutes, and the supernatant discarded. Prior to DNA extraction, extracellular DNA was depleted through 1 x round of 1 µL of TURBO DNAse and 1 µL of RNAse Cocktail (Life Technologies Limited), the solution was incubated at 37 °C for 30 min. The DNAse and RNAse were inactivated using heat at 65 °C and 15 mM EDTA final concentration for 10 min. QIAGEN DNeasy PowerLyzer PowerSoil DNA Isolation Kits (Geneflow Limited, Lichfield, UK) was used as per manufacturers instructions. Kit negatives were processed with each batch. All DNA samples were stored at -80 °C.

Genome sequencing

Bacterial Community amplicon sequencing and analysis: The V4 region of the 16S rRNA gene, was used as a target for amplicon sequencing using the approach detailed by Kozich et al. (2013) (NU-OMICS, Northumbria University at Newcastle, UK).

Viral Community sequencing and analysis: The Viral DNA samples were prepared to libraries using the Illumina Nextera XT (Illumina, Saffron Waldon, UK) kit. A V3 600 bp kit was used for the loading and running of the sample. The DNA samples were diluted to 0.2 ng/μL (Qubit 2.0 DS HS DNA Kit, Life Technologies Limited). The Illumina Nextera XT library preparation kit was used as per manufacturers instructions. The samples were normalised using Illumina based bead solution. The samples were pooled and loaded onto the MiSeq. The quality of the run had a Q30 score of >75%. This generated paired end FASTQ files (NU-OMICS, Northumbria University at Newcastle, UK).

Data clean-up and read count/distribution

The sequenced reads were adapter trimmed and quality filtered using Trimmomatic (Bolger *et al.*, 2014) and Sickle (Joshi & Fass, 2011) respectively.

Bacterial and fungal operational taxonomic units (OTU's) were defined using the OptiClust method in Mothur (Kozich *et al*. 2013). Taxonomy for each OTU was assigned using the Silva database for bacterial OTUs and the UNTIE database for fungal OTUs .

Viral shotgun sequencing reads were concatenated and converted from fastq to fasta format. The raw reads were blastn searched (Altschul *et al.*, 1990) against a preformatted NCBI database using word size 20 and percentage identity 70. For the viral analysis the pre curated viral database was downloaded and used as a reciprocal viral database.

Megan 6 was used to analyse the blast output file (Huson *et al.*, 2007) where lowest common ancestor and relative abundance was calculated from the data. The sample groups were then loaded for comparison and normalized by count and assessed based on genus rank.

A program tool was built to identify fold change and p values for every taxa identified (10's of thousands), which it then further cross-compared and filtered within the program to statistically identify taxa of interest. This was necessary due to the PCoA approach to visualizing the data as a whole.

A second program was created 'ViralHostFinder', which was used to find the related bacterial host of each viral taxa identified.

Sequence counts were normalised by conversion to relative abundance for single-omic analyses (i.e. comparing two bacterial communities), or cumulative sum-scaling followed by centered log-ratio transformation for multi-omic analyses (i.e. comparing combined bacterial, FVP and IV communities).

Sequencing output parameters

A total of 1.79×10^4 viral sequencing contigs, assembled from shotgun sequence reads, mapped to 231 taxa within the NCBI viral database. These sequencing contigs were spread across FVP (8.95×10^3), and IV (8.96×10^3) communities. Mean sequencing contigs per sample were 186.66 ± 36.65. Targeted bacterial 16S rRNA gene sequencing yielded 3.43×10^6 sequence reads from 8.82×10^3 bacterial taxa following removal of control samples from analysis. Mean reads per sample was $7.14 \times 10^4 \pm 2.30 \times 10^4$. Fungal communities yielded very low sequence reads per sample (mean 98.55 ± 278.70), and as such these communities were not included in further analysis.

Classification of responders and non-responders to dietary periodisation

Study participants were classified as either responders' or non-responders based on the impact each dietary intervention had on time trial to exhaustion performance. A linear regression model was built based on the overall response of each participant to the relevant periodisation diet. Because HCD generally improved performance HCD responders were defined as those whose performance at TTE *improved* more than would be estimated by the linear regression model. In contrast, HPD generally reduced performance, therefore responders to this diet were defined as those whose performance at TTE *reduced* more than estimated by the model.

Grouping functional genes by functional category

VOG annotated genes are already classified as either viral structure or replication genes. We further classified those without any previous classification in to one of 9 high level functional groups according to

string searches performed on assigned Consensus Functional Descriptions of VOGs as outlined in the table below:

Functional group	*Associated strings*	*VOGs count*
DNA/RNA processes	DNA, RNA, Resolvase, Helicase, Polymerase, Primase, Transcription, Topoisomerase, Replicase, Endonuclease, Exonuclease,	566
Host metabolism	Phophatase, Transferase, Esterase, Hydrolase, Reductase, Kinase,	366
Host lysis	Lysin, Holin, Tubin	104
Host infection	Recombinase, Integrase, Excisionase, Terminase, protein alc, prr	182
Viral Structure	Tegument, Head, Tail, Baseplate, Tail fibre, Capsid, Spike, Structural, Envelope, Virion, Decoration, Scaffolding, Matrix, lipoprotein, glycoprotein	999
Viral replication	rIIa, rIIb, Deoxytidylate deaminase, Replication	84
Putative/hypothetical protein	Putative, Hypothetical	24380
Uncharacterised	Uncharacterised	792

All genes assigned Consensus Functional descriptions that did not match any of the strings listed above were classified as 'Other' (886 genes).

<u>References</u>

Altschul, S. F. et al. (1990) 'Basic local alignment search tool', Journal of Molecular Biology, 215(3), pp. 403–410. doi: 10.1016/S0022-2836(05)80360-2.

Bolger, A. M., Lohse, M. and Usadel, B. (2014) 'Trimmomatic: A flexible trimmer for Illumina sequence data', Bioinformatics. doi: 10.1093/bioinformatics/btu170.

Harris, J. A. and Benedict, F. G. (1918) 'A Biometric Study of Human Basal Metabolism', Proceedings of the National Academy of Sciences, 4(12), pp. 370 LP – 373. doi: 10.1073/pnas.4.12.370.

Henry, C. J. K. (2005) 'Basal metabolic rate studies in humans: measurement and development of new equations', Public Health Nutrition. 2007/01/02. Cambridge University Press, 8(7a), pp. 1133–1152. doi: DOI: 10.1079/PHN2005801.

Huson, D. H. et al. (2007) 'MEGAN analysis of metagenomic data', Genome Research. doi: 10.1101/gr.5969107.

Joshi, N. and Fass, J. (2011) 'Sickle: A sliding-window, adaptive, quality-based trimming tool for FastQ files (Version 1.33) [Software]', Available at https://github.com/najoshi/sickle.

Kozich, J. J. et al. (2013) 'Development of a Dual-Index Sequencing Strategy and Curation Pipeline for Analyzing Amplicon Sequence Data on the MiSeq Illumina Sequencing Platform', Applied and Environmental Microbiology, 79(17), pp. 5112–5120. doi: 10.1128/Aem.01043-13.

Tariq, M. A. et al. (2015) 'A metagenomic approach to characterize temperate bacteriophage populations from Cystic Fibrosis and non-Cystic Fibrosis bronchiectasis patients', Frontiers in Microbiology. Frontiers Research Foundation, 6(FEB). doi: 10.3389/fmicb.2015.00097.
