# Supplementary material for: Gut Microbial Stability is Associated with Greater Endurance Performance in Athletes Undertaking Dietary Periodization
Source: mSystems. 2022 May 17;7(3):e00129-22. doi: 10.1128/msystems.00129-22 (PMC9238380; doi:10.1128/msystems.00129-22)
Supplement: TABLE S2 [file msystems.00129-22-s0007.pdf]

Supplementary table 2 – Adonis PERMANOVA results: gut microbial beta-diversity

Dynamic changes in the gut microbiota in response to acute high protein and high carbohydrate diets in endurance athletes.

Furber, M.J.W., Young, G.R., Holt, G., Pyle, S. Howatson, G., Roberts, M.G., Roberts, J.D. and Smith, D.L

	BACTERIAL		FVP		IV	
	R^2	P	R^2	P	R^2	P
**Adonis PERMANOVA**						
Variables						
Patient	0.80	0.001*	0.51	0.001*	0.52	0.001*
Diet : Time point	0.03	0.216	0.10	0.001*	0.10	0.009*
Residuals	0.17	-	0.38	-	0.38	-
Time points by diet						
EARLY vs MID vs LATE (HCD)	0.03	0.397	0.07	0.192	0.06	0.459
EARLY vs MID vs LATE (HPD)	0.03	0.241	0.14	0.002*	0.13	0.009*
**pairwise Adonis PERMANOVA**						
Carbohydrate						
EARLY vs MID	-	-	-	-	-	-
EARLY vs LATE	-	-	-	-	-	-
MID vs LATE	-	-	-	-	-	-
Protein						
EARLY vs MID	-	-	0.15	0.023*	0.16	0.016*
EARLY vs LATE	-	-	0.04	0.351	0.16	0.781
MID vs LATE	-	-	0.10	0.110	0.10	0.102
